# Stainless steel weld metal enhanced with carbon nanotubes

**DOI:** 10.1038/s41598-020-75136-z

**Published:** 2020-10-21

**Authors:** D. J. A. Borges, D. C. S. Cardoso, E. M. Braga, A. A. F. Castro, M. A. L. Dos Reis, C. R. L. Loayza

**Affiliations:** 1 Programa de Pós-Graduação em Engenharia Mecânica (PPGEM/UFPA), Universidade Federal do Pará, Belém, PA 66075-110 Brazil; 2 Programa de Pós-Graduação em Engenharia de Recursos Naturais da Amazônia (PRODERNA/ITEC), Universidade Federal do Pará, Belém, PA 66075-110 Brazil; 3 Faculdade de Ciências Exatas e Tecnologia, Universidade Federal do Pará, Abaetetuba, PA 68440-000 Brazil

**Keywords:** Nanoscience and technology, Nanoscale materials, Other nanotechnology, Techniques and instrumentation, Mechanical engineering, Nanoscale materials, Techniques and instrumentation

## Abstract

This paper aims to establish the most indicated route to manufacture a nanostructured powder composed of 5 wt% Multi-walled Carbon Nanotubes and 304LSS powder. Four specimens were prepared using Mechanical Alloying and Chemical Treatment (CT) with Hydrogen Peroxide (${\mathrm{H}}_{2}{\mathrm{O}}_{2}$) as the main processes. A thermal treatment post-processing was used in half of the samples to remove the remaining amorphous carbon and to evaluate its effects. Regarding the powder analysis, attachment, amorphous carbon degree, crystallinity, and doping of the CNT throughout the metal matrix were investigated. The nanostructured powders were then inserted as a core in a 304LSS tubular rod to perform the arc welding process. The CT route eliminated the amorphous carbon and generated more refiner grains, which provided a cross-section hardness gain of more than 40% regarding the 304LSS joint. In summary, the CT route, combined with the GTAW process, provided a new method for nanocomposite manufacturing by combining shorter preparation steps, obtaining an improvement in the microstructural and hardness performance.

## Introduction

The carbon nanotubes (CNT) are a synthetic allotropic form of carbon, composed of a graphene sheet rolled into a cylindrical shape. Since its discovery by IIJIMA, the scientific community seeks different ways of using their unique characteristics to incorporate the CNTs in metal matrices. Due to its C–C bonds and an *sp*^2^ hybridization, the CNTs have a higher tensile strength than Kevlar (100 GPa), a Young Modulus of 1.28 TPa, and also an elevated thermal conductivity of about 2000 ${\mathrm{W m}}^{-1}{\mathrm{K}}^{-1}$, more than 500% higher than copper and 800% than aluminum^1,2^. Despite these outstanding properties, factors such as the Van der Waals forces, which operate at the walls of the nanotubes, manufacturing limitations that may result in CNTs with a poor crystallinity, and its insolubility in most solvents are still some of the shortcomings to be faced^3,4^.

To overcome the aforementioned issues, both mechanical and chemical techniques were developed to manufacture nanostructured powders. As for the former, the Mechanical Alloying (MA) stands out because it is a method in which the CNTs and the powdered metal matrix are inserted in a ball mill along with steel spheres, which are rotated in a determined velocity to allow the friction energy that is generated to cold-weld the nanotubes on the surface of the metal matrix^5^. It is a relatively inexpensive process, and preserves the involved materials in the solid-state, avoiding possible phase limitations^6^. This process has been used to aggregate CNTs into metal matrices such as silicon^7^, copper^8^, aluminum^9,10^, and nickel^11^. Although the MA is an advantageous method, it inherently causes damage such as strains in the structure of the CNTs due to the repeated impacts of the steel balls and the discrepancies between the morphology of both nanomaterial and the metal matrix, which can even modify the hybridization of the nanomaterial. Therefore, parameters such as milling time, size and quantity of the spheres, atmosphere and pressure inside the mill, and the ball-to-powder ratio must be rigidly controlled.

Therefore, the selective oxidation method rises as an alternative for the MA process since it is a chemical treatment that breaks the Van der Waals forces, therefore causing some surface modifications in the hexagonal lattice of the CNTs and a possible bond of functional groups (functionalization)^12–15^. The oxidant reagents that allows this effect to take place can be Nitric Acid (HNO_3_)^16–18^, Hydrochloric acid (HCl)^19^, Sulphur acid (H_2_SO_4_)^20^, Sodium Hydroxide (NaOH)^21^ and Hydrogen Peroxide (H_2_O_2_)^22–25^. The latter is extensively used in the surface modification of carbon nanotubes due to some outstanding properties, such as being a mild reagent, i.e., barely damaging the hexagonal lattice. It is used as an iron catalyst without incorporating any foreign metals into the carbon surface and is also environmentally friendly when used in CNTs, as its main reaction product is water^26–28^.

Both MA and CT can be used to fabricate nanostructured powders, thus being used welding processes, highly used in the industry, and therefore, alternatives to manufacture nanostructured composites. The Gas Tungsten Arc Welding (GTAW) is noteworthy due to a greater operational control, few defects if related to other processes, no need for cleaning, it can be used to weld all metals, greater welding quality and low cost. The Pulsed one (P-GTAW) generates finer grains, increasing the performance of the weld metal, and contributes with the dispersion of elements alloys, which supports the fabrication of nanocomposites as it helps to avoid the vaporization of the nanomaterials and improves its dispersion in the weld metal. The GTAW has been used to manufacture nanostructured composites with Al/TiC^29^, Nickel-CNT^30^, and Al-CNT^31^ as additional material. Although several metal matrices were incorporated in a weld joint, researches involving stainless steel (SS) are still underdeveloped, as their high melting point is very destructive for the structure of the CNTs. Despite that, as they are extensively used in industrial processes due to its alloying elements, such as nickel, chromium, and silicon^32^, its use in Metal Matrix Nanocomposites (MMNCs) becomes vital. The SS 300 series stands out due to its high ductility, which mitigates the residual stresses generated during the welding processes. The 304LSS is the most common one used in the market due to its excellent cost–benefit regarding mechanical properties, even though some limitations as void swelling, low thermal and electric conductivity must be properly addressed. The successful manufacturing of a 304LSS-CNT nanostructured composite via P-GTAW was reported by Loayza et al., in which a 30% improvement of lateral hardness if compared to the pristine SS was attested.

Regarding the welding process with carbon nanotubes, to control the crystallinity level of the nanomaterial with the amount of amorphous carbon in the samples becomes imperative. The presence of such components facilitates the formation of chromium carbides in the stainless steel, which is responsible for the sensitization effect, intergranular corrosion caused by the precipitation of these on the grain boundary during steel cooling, in temperatures between 1200 and 750 °C^33^. The Chromium carbides act as cathodic sites to the chrome-depleted neighboring matrix, leading to a corrosion process located in the region between the material grains, subsequently causing mechanical fragility^34,35^.

**Figure 1:**
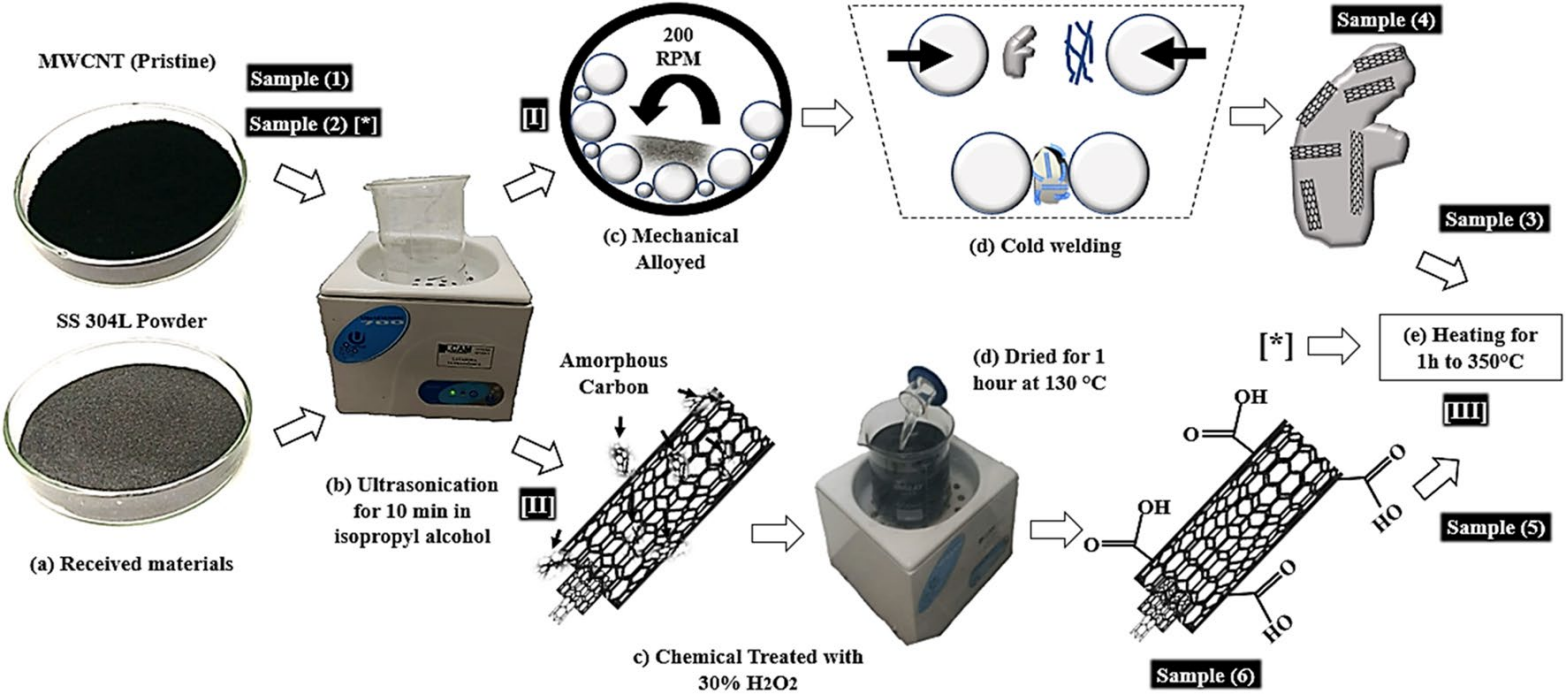
Schematic of the manufacturing routes of the 304LSS-CNT powders. The three routes analyzed, [I], [II], and [III], with thermal treatment for each sample. (**a**) As-received materials, 304LSS and 5wt.% MWCNT, (**b**) Ultra sonication for 10 min in isopropyl alcohol, (**c**) MA and CT with H_2_O_2_, (**d**) Cold weld and dried for 1 h, and (**e**) Heating for 1 h to 350 °C.

The present report aims to establish the best route to fabricate nanostructured composites of 304LSS as metal matrix and Multi-Walled Carbon Nanotubes (MWCNTs) as a core in a P-GTAW process. The best parameters to produce a nanostructured powder via both CT and MA, regarding the best dispersion and aggregation conditions, were evaluated, as well as the higher hardness resistance and the least time-consuming preparation process for the nanostructured composite as the final product. The methods to disperse the nanotubes and further aggregate in the metal matrix were the Mechanical Alloying, chemical treatment with Hydrogen Peroxide, and thermal treatment. Figure 1 shows the methodology to obtain the six samples used, which are further described in the Experimental Procedure section.

## Results and discussion

### Nanostructured powder characterization

**Figure 2:**
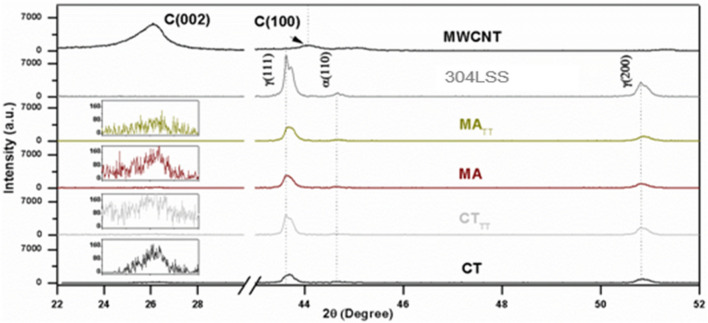
XRD patterns for the CNT as received, as well as the 304LSS metal matrix and the nanostructured composite with their respective processing routes.

Figure 2 shows the XRD characteristic spectra related to the crystallographic planes of the analyzed samples, between the angles 0° to 60°. In the specimen of pure carbon nanotubes, the typical peaks to the nanomaterial graphite structures can be noticed, both (002) at approximately 26.3°, that refers to the longitudinal structure of the CNTs, and (100) at approximately 44.7°, representing the tips of the nanomaterial^36–38^. The 304LSS metal matrix signature shows the planes referring to the austenitic structure (face-centered cubic) in the (111) and (200) planes, at approximately 44° and 51°, respectively. The body-centered cubic structure, named as ferritic, in the (110) plane at approximately 45°^39,40^. Both planes are highlighted in the *as-received* sample indicates that the carbon nanotubes are mostly agglomerated before being aggregated in the metal matrix. As they are mixed, the most prominent peak of the MWCNTs is considerably suppressed due to the intensity difference between the former and the analyzed steel. Besides, it is noteworthy that there was a decrease in the (002) spectra of the graphitic structure of the CNTs after the Thermal Treatment, probably indicating that the nanomaterial is more dispersed and loosely attached to the MM throughout the sample.

Although the characteristic peaks of the nanotubes were not highlighted in the nanostructured powders, its influence in the metal matrix can be noticed as the 304LSS signature was remarkably flattened in all the analyzed samples. This is due to the capacity of the CNTs to absorb X-Ray, named as “X-Ray attenuation”, as carbon behaves similarly to a blackbody, which absorbs all wavelengths of light, and the inherent properties of the nanotubes, such as high specific surface area, higher conductivity, high strength and promising electromagnetic properties, if compared to traditional materials^41,42^. Regarding the metal matrix, the (111) austenitic phase suffered the most evident XRD attenuation; if compared to the pristine sample (304LSS), the CT_TT_, MA_TT_, MA and CT specimens, respectively, had approximately 57.7%, 66%, 66.83% and 80.48% of X-Ray intensity reduction.

In order to further understand the influence of each route, through which the nanocomposite powders were fabricated, the specimens were characterized with Raman Spectroscopy between 1250 and 1700 cm^−1^ for the MWCNT specimens as received (a,b), the nanostructured powder subjected to the Mechanical Alloyed route (c,d), and the chemically treated ones (e,f), as shown in (Fig. S1). The disorder-induced D-Band^43^ and the amorphous carbon indicator ${D}_{middle}$ (or D”)^44^ were both fitted with one Lorentzian peak each. The Fig. S1d sample, however, presented a satellite peak named as S, that corresponds to a solvent-free functionalization caused by the milling process, in which the energies involved during the repeated impact of the steel balls cause distortions in the crystalline structure of the CNTs, rearranging some of the broken *sp*^2^ bonds into *sp*^3^ ones^45,46^.

As for the first order G-band, that corresponds to the *sp*^2^ tangential vibrations of the carbon atoms, and it is commonly associated with the CNTs crystallinity^47^. All the analyzed samples were fitted with four peaks each. ${G}_{BWF}$, indicating a metallic behavior in the MWCNTs^45,48^. G_inner_, which is linked to the tangential vibrations of the inner tubes^49,50^. G_outer_, always located after G_inner_, is associated with the tangential vibrations of the outer tubes, and with a less intense signature due to defects in the hexagonal lattice, as they are more exposed to the nanotubes surroundings^49^. D′, an overtone of the second-order D-band, also associated with defects in the MWCNTs structure^51^. The supplementary material also presents the amplitude and center position values of the bands and sub-bands.

The analysis of the pristine samples, examining the D-band, indicates that the thermal treatment itself did not cause defects in the MWCNTs, but cleaned the inactive carbons instead, which were probably evaporated during the heating process, as the literature states^52,53^. As for the S-peak in the MA specimen, the thermal treatment was responsible for exfoliating the outer tubes, resulting in a 62% increase of the second-order band, that is, severe degradation of the MWCNTs structure. The chemically treated nanostructured powder presented a 56% intensity reduction of the D-band, suggesting that the cleaning that took place in the pristine samples has also occurred in this case. G-band and its sub-bands increased the crystallinity for both the pristine specimen, among the thermal treatment (TT), with a G_outer_ enlargement of approximately 127%, and a 16% raise of the MA sample regarding the non-Thermally Treated one. The Chemically Treated samples, though, presented a 62% intensity decrease of G_outer_ after the TT, indicating a substantial crystallinity loss due to the damage caused in the outer walls of the MWCNTs by the selective oxidation process^54^.

**Figure 3:**
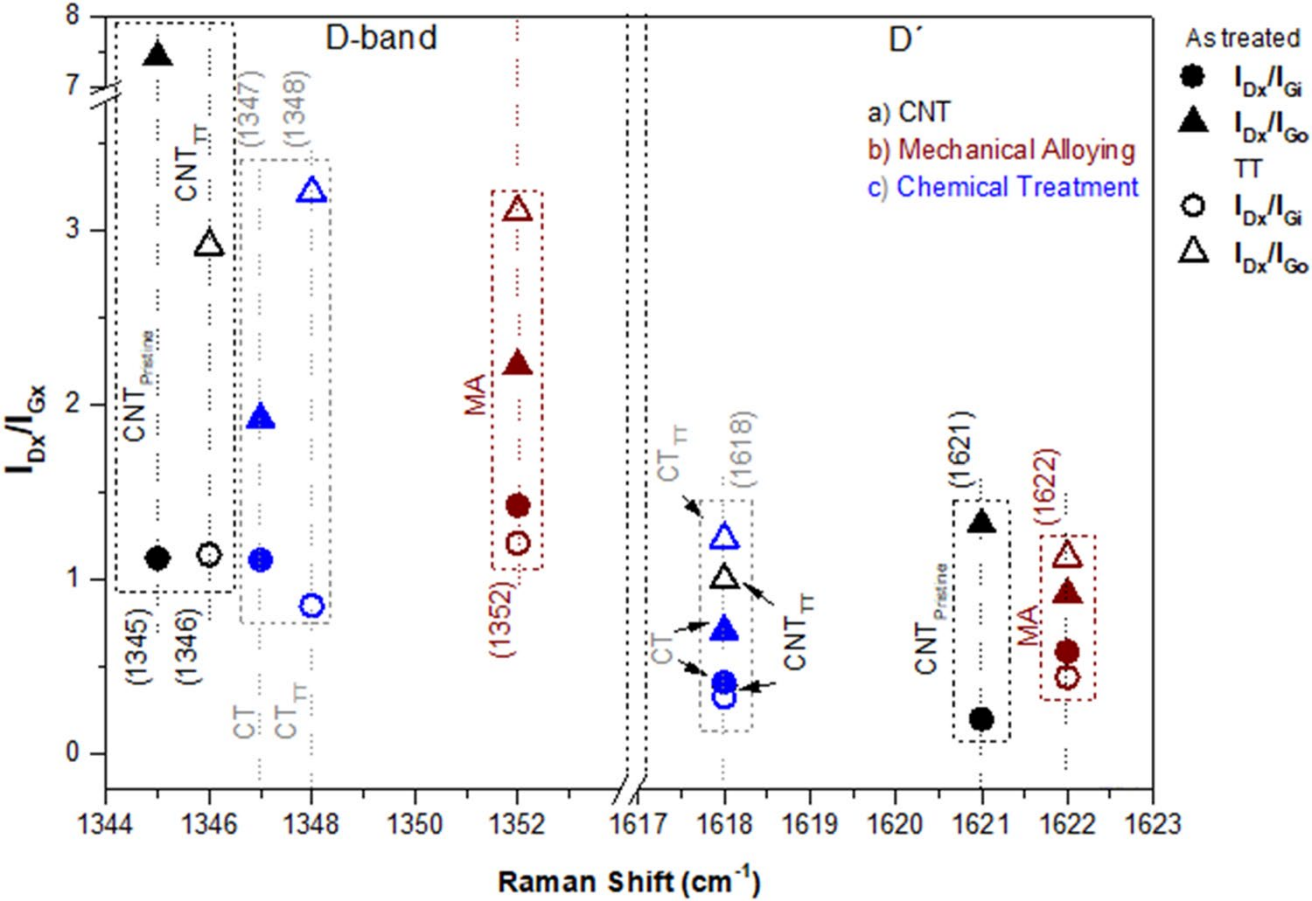
Intensity ratios of peaks: first order (D and D′) where the I_x_ corresponds to intensities for each Raman band to the various samples.

Figure 3 presents the I_D_/I_G_ defect degree ratio^55^ for both the inner (I_D_/I_Gi_) and outer (I_D_/I_Go_) walls of the MWCNTs, as well as the I_D′_/I_G_, another defect ratio parameter of the hexagonal lattice of the nanotubes^51^. It can be noticed that, if compared to the outer walls of the MWCNTs, the inner ones suffered minor modifications in the I_D_/I_Gi_ ratio after the thermal treatment, i.e., approximately 1% increase in the pristine sample, 15% decrease for the MA sample, and a 24% decrease of the level of defects of the CT. As for the outer walls, the influence of the TT can be better scrutinized, as they are more exposed to the environment. The I_D_/I_Go_ ratio before and after the TT process indicated a reduction of about 60% of the as-received nanotubes, and 40% in the MA route and 67% in the sample submitted to the H_2_O_2_ selective oxidation.

Although the Thermal Treatment mildly reduced the level of defects in the inner walls of the samples, it is noteworthy that the nanotubes purification process occurred distinctively between the routes, as the intensity values presented in Table S5 suggest. By analyzing the MA one, the level of defects was reduced due to a significant influence of the graphitization of the G_i_ sub-band, with an increase of about 92% after the Thermal Treatment, more significant than the 62% of the D band, possibly generated by the increased damage of the hexagonal lattice caused by the repeated impact of the steel balls during the milling process. As for the Chemical Treatment route, the G_i_ sub-band suffered a 19.4% crystallinity decrease, indicating that the selective oxidation process affects the whole structure of the nanotubes, being more susceptible to structural damage^53^. However, the D-band also decreased by 50%, demonstrating a significate reduction of impurities inlaid in the MWCNTs, which possibly reacted to the hydrogen peroxide and evaporated through heating.

To the pristine sample, the TT process was responsible for a considerable cleaning effect of the impurities presented in the hexagonal lattice, relating to the analysis of the level of defects of the most external walls. For the subsequent samples, there was a severe loss in the crystalline structure of the CNTs, as Table S5 intensity values indicate. In the MA route, the I_D_/I_Go_ ratio increase is mainly due to the 60% growth of the D-band, indicating that the oxidation process was intensified as a result of the structural damage caused by the ball milling, overcoming the modest 16% increase of the G_o_ sub-band. This has positively affected a part of the hexagonal lattice submitted to the TT that was not harmed by the steel balls. Regarding the CT specimen, the level of defects was arisen by the 160% crystallinity loss of the outer walls, a result of the H_2_O_2_ reaction with the high temperature of the TT, showing that the solution is still responsible for causing defects in the hexagonal lattice of the nanotubes when exposed to a considerable amount of time and/or temperature, despite being a mild solution if compared to the acid reagents^12,28^.

**Figure 4:**
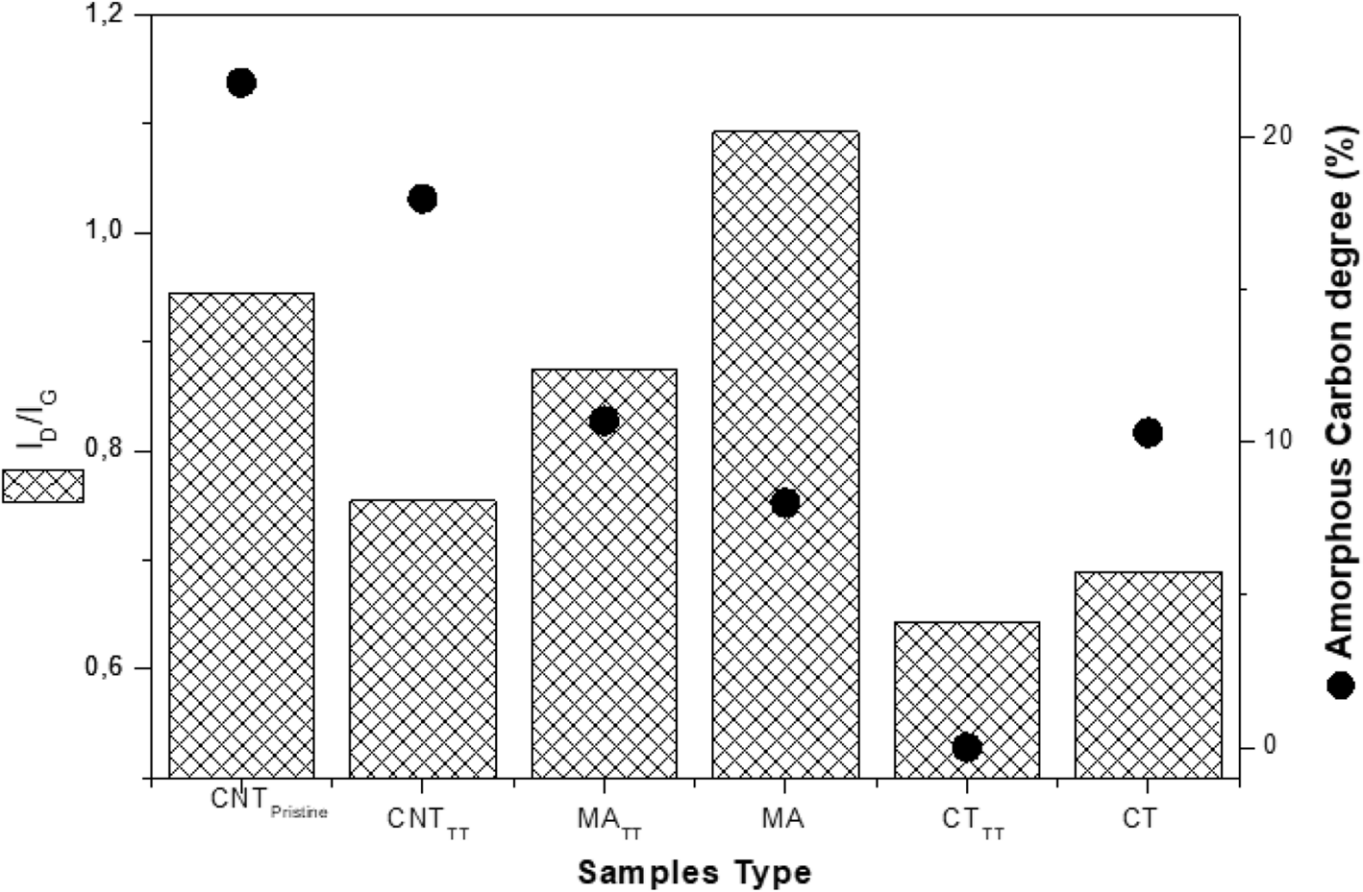
Amorphous carbon degree of each analyzed sample.

As seen in the Fig. S2, in all types of treatment a blueshift in the I_D_-band first mode was noticed, mainly in the mechanical allowing ones (12 cm^−1^). In the second mode, both D′ thermal and chemical treatments had a redshift while the mechanical alloying ones had a blueshift, suggesting a different behavior involved the kind of treatment applied and the resonance mode. When the CNTs were damaged by the milling process^56^, they were shortened and produced breakage regions located in their tips^57^, as corroborated in the MET images (Fig. S7). Figure 4 presents the graphs of the Amorphous Carbon Degree (ACD)^45^ values for the analyzed samples and the level of defects (I_D_/I_G_) for the MWCNT as a whole. It can be noticed that there was a reduction of the ACD after the thermal treatment, for both the pristine CNTs and the CT one, indicating that these were partially eliminated, either by simple evaporation or by the formation of carbon monoxide and dioxide when bound with the oxygen, generated from the hydrogen peroxide that reacts with the nanotubes^13^. Regarding the MA specimen, ACD slightly increases after TT, although the level of defects has been reduced. This can be explained by the amorphization of part of the crystal lattice of the MWCNTs that was harmed during the ball milling process. Regarding the I_D_/I_G_ ratio, it is evident that the thermal treatment plays a significant role in removing the defects of the MWCNTs. After the TT, there was a reduction of 20.07%, 19.88%, and 6.82% in the CNT as-received, MA, and CT specimens, respectively. It is worth noting that, despite a remarkable increase of the I_D_/I_Go_ of the CT_TT_, if compared to the non-treated CT, there was a modest decrease in the level of defects regarding a broader evaluation of the MWCNTs, that indicates a higher removal of impurities on the inner walls of the carbon nanotubes, as the I_D_/I_Gi_ ratio indicates (Figs. S8, S9, S10).

The second-order modes (2D-band) intensities, shown in the Fig. S3, were affected significantly with the type of treatment. The intensity I_2Dr_ (Table S5) shows changes in the behavior of the outer sidewalls of the CNTs with the various process, the higher intensity was the CT (187), almost 68% respect to the CNT pristine, following by the CNT_TT_ (147). The 2D_l_ peaks were only observed in the CNT_TT_ (**I**_**2Dl**_**/I**_**Gi**_ = 0.43) and CT (**I**_**2Dl**_**/I**_**Gi**_ = 0.23) samples, demonstrating that there is a minimal interaction in these types of treatment, of the internal sidewalls that do not appear in the other samples. The ratio intensities were I_2Dr _∼ 0.82I_2Dl_ and for the second I_2Dr_ ∼ 3.17I_2Dl_, respectively. This indicates that CNT chemical treatment has a substantial electron charge transfer of the external sidewalls with the 304LSS powder. CNT_TT_ had elimination of the amorphous carbon and purification of the external sidewalls, resulting in a higher interaction of the inner sidewalls with the outer itself, and their neighborhoods^27^. To the CNT_Pristine_ existed other peaks labeled as DD_l_ (*), 2D (**) and DG_l_ (***)^58^, which might indicate an interaction with the outer sidewalls by their proximity, instead of with the inner ones. In other cases, the effect of the inner ones through the D_l_ is minimal.

As displayed in the Fig. S4, the linewidth peaks (2D_l_ and 2D_r_-bands) regarding the CNT_TT_ and CT were 28 and 32 cm^−1^, respectively, indicating stress levels that could be associated with inherent deformations presented in the CNTs that were more evident with both treatments. The FWHM of the 2D_r_-band of the CNT_TT_, MA_TT,_ CT, MA, CT_TT_, and CNT_Pristine_ were 56, 59, 60, 66, 67, 72, respectively. All treatments reduced the 2D_r_-band FWHM; as for the TT, the reduction was more significant, followed by MA_TT_ and the CT. The MA and CT_TT_ had the lowest ones. The former by the increment of the amorphous carbon and functionalized group derivate of the mechanical process, shrinking the CNT and multiplied the number of defects in the edges^56,57^. The latter, the effect of the two treatment (CT and TT) affected the integrity of the walls, multiplied the defects number, and removed the possible polycyclic aromatic hydrocarbon structures formed by the H_2_O_2_ in the chemical treatment^28^, reducing the interaction with the metal particles. Table S5 shows a redshift in each treatment, indicating higher stress levels coming from the tensile strain. The redshift of the G_outer_ in comparison with the pristine ones was 11, 12, 9, 11, and 18 cm^−1^ to the CNT_TT_, MA_TT_, MA, CT_TT_, and CT, respectively. Each treatment had n-doping influence^45^ in the CNT, highlighting the CT with a strong interaction derivate of the functional groups formed by the H_2_O_2_ reaction^24,59^. The G_inner_ had similar behavior with a redshift of 3, 2, 1, 4, and 7 cm^−1^. The two last had higher values of n-doping, showing the strong interaction with the matrix; this could have favored the better results in the microhardness of both treatments.

Figure S2a shows that the CNT_Pristine_ has a vast difference between the FWHM of the G_inner_ and G_outer_ by the high amorphous carbon and impurities content. The treatments influenced in the linewidth of the two peaks. The relation between the I_Gouter_/I_Ginner_ indicates that the MA and the CT have the strong charge transfer (f). As displayed in the Fig. S2b, the FWHM for the outer tubes increased 23.58 cm^−1^ and 22.99 cm^−1^, respectively (f is 0.05 holes per carbon atom for both). To the inner tubes, the FWHM decreased 34.62 cm^−1^, obtaining an f of 0.07 holes per carbon atom. The G_BWF_ is observed in all the cases, it changes their position (1554–1576 cm^−1^) and intensity, indicating n-doping of the innermost tubes to the outermost due to the charge transfer^45^, that occurs by the tensile strain due to bulking, bending and compression, as observed in the TEM images. The higher intensities were both the CNT_TT_ and CT, which was confirmed by the 2D_l_ intensity presence. D + G mode only was observed in the CT, CT_TT_, and mechanical alloying, which indicates high interaction between the CNT and MM.

**Figure 5:**
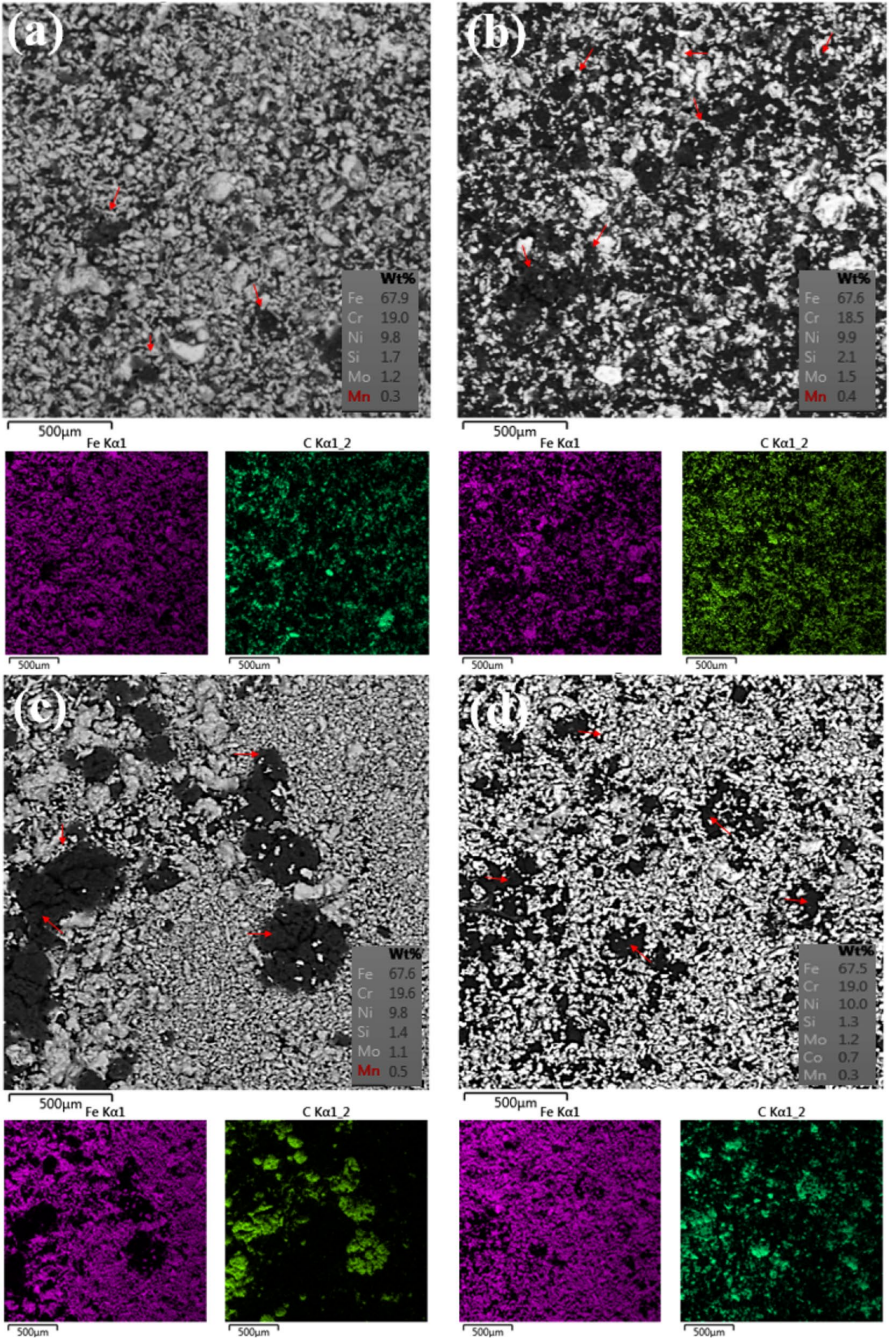
SEM images of the (**a**) after ultra-sonicated (**b**) Mechanically alloyed and chemically treated nanostructured powders (**c**) with and (**d**) without TT; the red arrows indicate CNT bulks.

Figure 5 presents both SEM and EDS images of the CNT-304LSS nanostructured powders after ultrasonication (Fig. 5a), MA, (Fig. 5b), and CT_TT_ (Fig. 5c) and CT (Fig. 5d), with red arrows indicating the CNT clusters throughout the samples. By comparing with Fig. 5a, the MA sample exhibited more dispersed MWCNTs along with the analyzed frame, as it can be noticed by the carbon signature of the EDS mapping in the latter, with significantly fewer dark spots than the former. Despite that, as the MA process does not permanently weaken the tube-tube Van der Waals forces, some clustered nanotubes were still present in the sample. The H_2_O_2_ effectiveness in dispersing the CNT can be visualized in Fig. 5d; although some dark spots are still perceived in the carbon EDS mapping, by the analysis of the SEM image, the chemically treated sample without TT showed the best results, as barely any clustered nanotubes were visualized in the sample. This demonstrates that the hydrogen peroxide indeed facilitates the breaking of the Van der Waals forces, which toughens the dispersion of the CNT. Figure 5c, on the other side, showed the worst results regarding the dispersion matter; some bulks of CNTs were formed throughout the sample, indicating that high temperatures damage the positive influence of the chemical treatment that was perceived in Fig. 5d. Figure S6 shows complementary images that support this.

**Figure 6:**
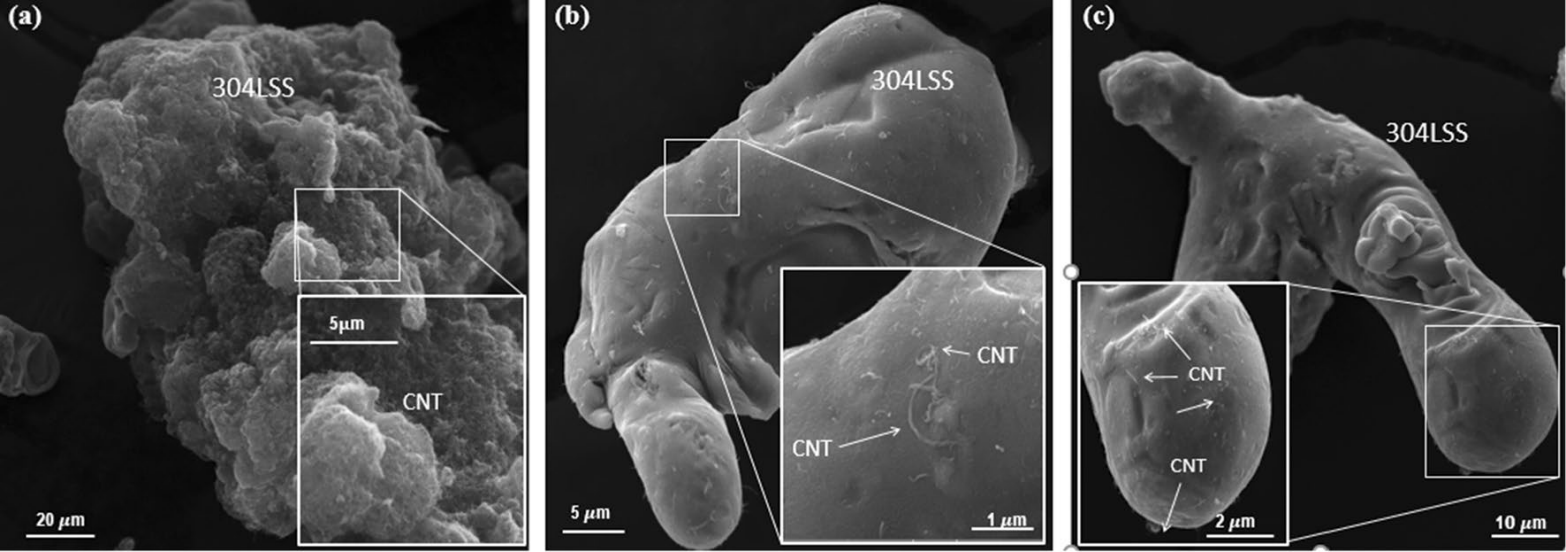
SEM images of the (**a**) MWCNT cluster (**b**) Mechanically alloyed and (**c**) chemically treated nanostructured powders.

Figure 6 presents SEM images of the after ultra-sonicated, mechanically alloyed, and chemically treated nanostructured powders, respectively. The isopropanol process itself, common to all samples, was not intense enough to weaken the Van der Waals bonds among the nanotubes, resulting in MWCNTs bundles covering the whole 304LSS matrix. This is not appropriate for the future manufacturing of a nanocomposite via welding, as there is a high probability that these bulks will generate discontinuities regarding the mechanical properties of the welded joints. On the other hand, for both the MA and CT specimens, it is clear that the nanotubes are more aggregated in the metal matrix, whereas in the former, there are MWCNTs that are visually different in length due to the high impact energies involved in the alloying process, which is responsible for the breaking of the nanomaterial.

Although Fig. 5d shows that the dispersion of the CNT in the metal matrix was the poorest among the analyzed samples, the aggregation state of the CT one is still appreciable. It can be explained by the fact that the CNT clusters in the pristine sample were widely spread among the metal matrix, resulting in images such as Fig. 6a, in which the 304LSS is wholly covered with CNT bulks, leading to a reduced aggregation level. Even though the CT_TT_ presented some unwanted bulks throughout the sample, as can be seen in Fig. 6c, there was an improvement in the aggregation state of the CNT, as aforementioned. Even though the chemical treatment was majorly removed, due to the high temperatures of the Thermal Treatment, some appreciable effects of the former can still be detected in the CNT-304LSS powder.

**Figure 7:**
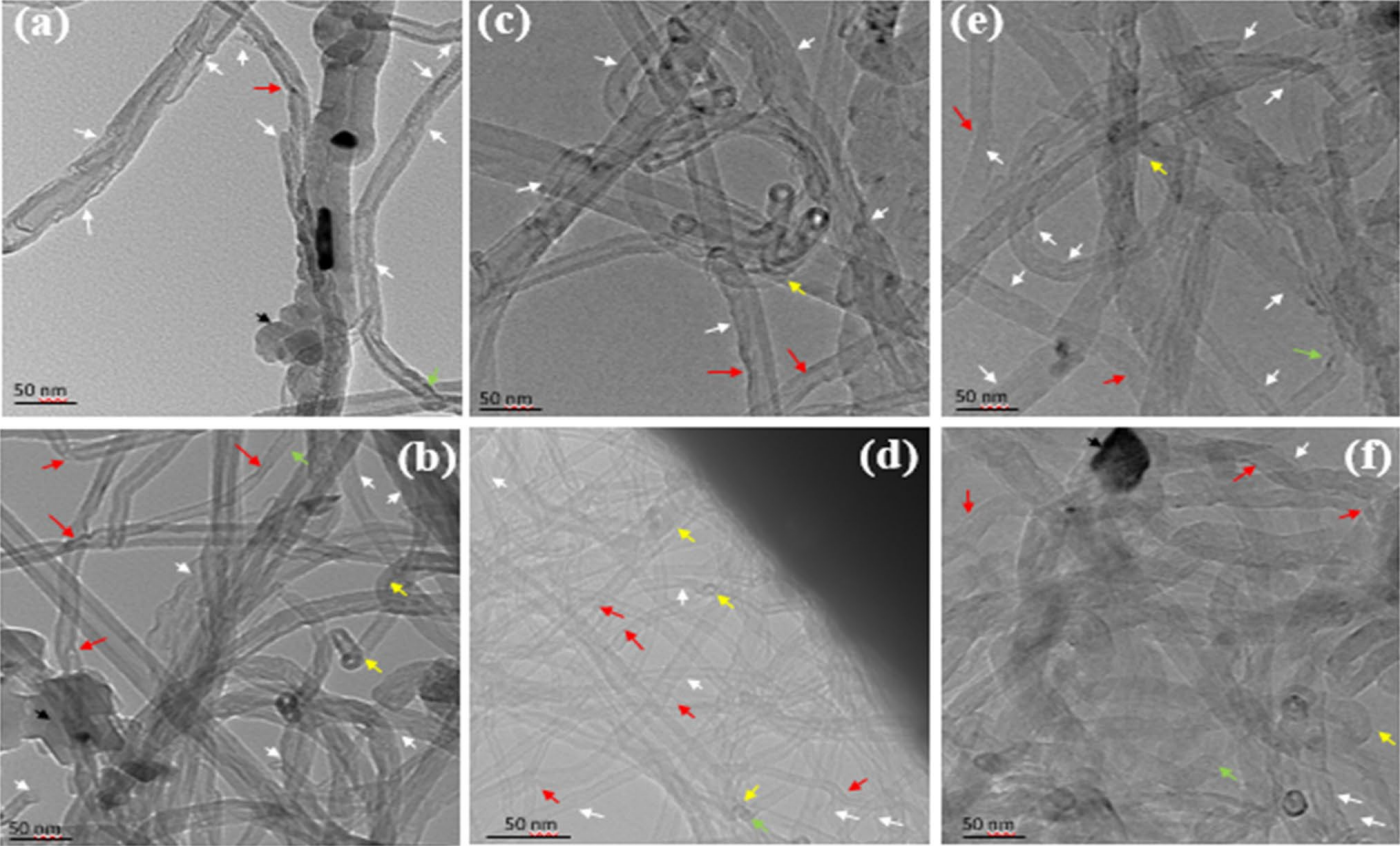
TEM micrograph of the CNT_TT_ (**a**), CNT as-received (**b**), MA_TT_ (**c**), MA (**d**), CT_TT_ (**e**), and CT (**f**). The TEM image allows the clear identification of several deformations such as exfoliation (white arrow), compression (red arrow), buckling (yellow), amorphous carbon (black), and tangling (green).

Figure 7 exhibits the TEM micrographs of the 304LSS-CNT powder. The sample Fig. 7a shows some catalysts residues^60^ and amorphous carbons (indicated by the dark arrow), and it is noticed that some exfoliation had already taken place during the ultrasonic bathing and the thermal treatment, as marked by the white arrows. By analyzing the MA micrograph (Fig. 7d), the damage done to the MWCNTs by the repeated impact of the steel balls is evident, as they appeared majorly tangled (green arrow), compressed (red arrow), buckled (yellow), and exfoliated; the latter was intensified by the thermal treatment (Fig. 7c), increasing the degradation of the outer walls of the nanotubes, corroborating with the Raman results. Although the MA sample presented the best results regarding dispersion, the MWCNTs in the CT process were the least damaged ones, as Fig. 7f shows. The nanotubes' integrity was preserved, with barely any compression or exfoliation, and low amorphous carbon. Figure 7e, on the other hand, shows that some of the MWCNTs were majorly harmed after the TT, as there was a significant intensification of the exfoliation process caused by the hydrogen peroxide, resulting in a loss of crystallinity that was previously discussed in the Raman results. Despite that, the bulk of amorphous carbon in Fig. 7f was removed from the sample because of the TT. The images from Figs. S7 to S10 support the aforementioned analysis.

The dispersion and aggregation state analysis of the nanotubes in the metal matrix indicated that there were little visual discrepancies when comparing the MA and CT routes. It is noteworthy that in the latter, despite a crystallinity reduction if compared to the former, the almost elimination of amorphous carbons was verified due to the Thermal Treatment, indicated by Raman analysis. It is noteworthy that the preparation time of the sample submitted to the CT was also significantly shorter than the Mechanical Alloying route, which facilitates its reproducibility.

### Nanocomposite characterization

**Figure 8:**
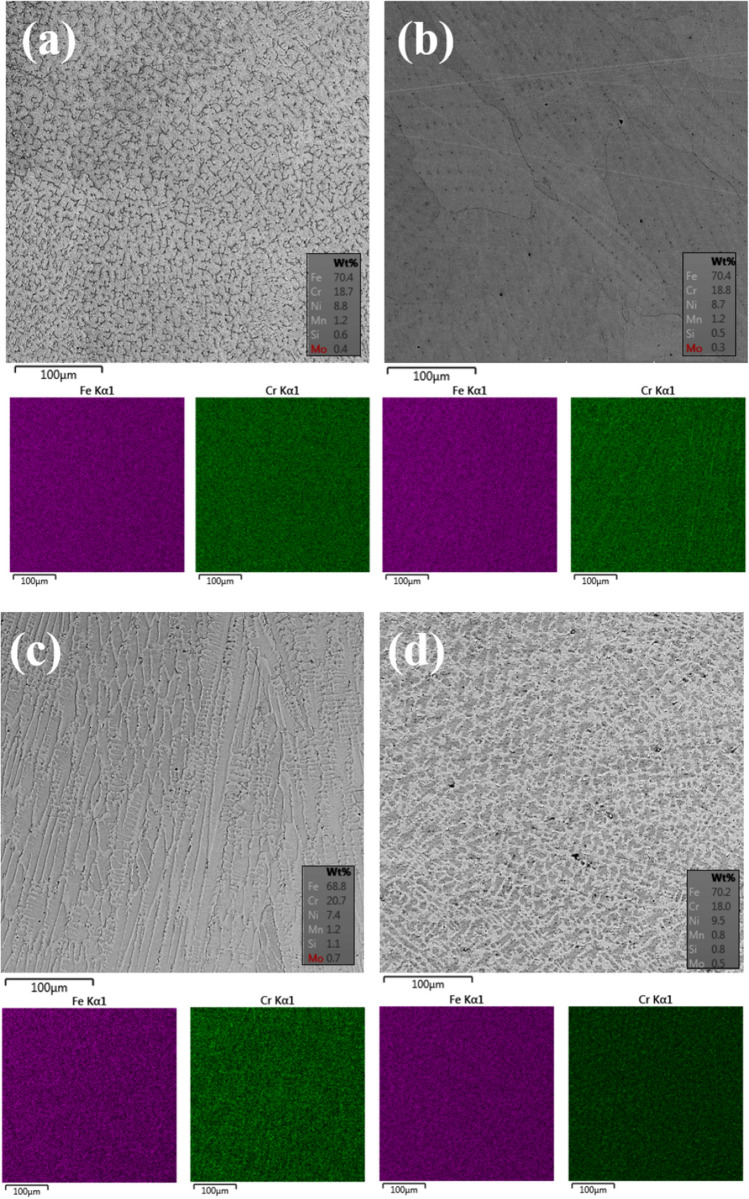
Microstructure of the metal matrix to (**a**) 304LSS, 304LSS-CNT with (**b**) MA, (**c**) CT_TT_, and (**d**) CT conditions.

Figure 8a present the microstructure of the welded 304LSS sample obtained via BSE, along with the EDS mapping of the Fe and Cr, presented in the joint. Figure 8b shows the microstructural aspects of the 304LSS-CNT nanostructured composite, submitted to the MA process. Figure 8c displays the microstructure of the 304LSS-CNT nanostructured composite that was chemically treated with H_2_O_2_ after TT, and Fig. 8d exhibits the CT sample. As it can be seen in Fig. 8a, the regular 304LSS structure is presented, i.e., an austenitic matrix with precipitation of vermicular ferrite^61,62^. It can be noticed by examining the nanostructured composites that the MWCNTs are majorly responsible for some considerable microstructural modifications. The analysis of the nanostructured composite that was processed via Mechanical Alloying indicates that there is a decrease of the ferrite phases and the grain boundaries are more susceptible to appear, like what has been described by Loayza et al. 2018. Although there was no substantial modification in the average values of the alloying elements of the nanostructured composite, some chromium spikes were detected, as seen in the supplementary material, indicating the possibility of carbide formation, which may also be responsible for an apparent grain refinement in the CT-TT sample^62^. By examining the EDS mapping, the chromium influence regarding the microstructural modifications becomes more evident, as there are substantially more spikes**,** increasing the chromium average, followed by a decrease in the iron quantities. It indicates that the formation of the carbides may become evident, as the grain boundaries are more distinguishable than the other samples. Even though there was barely any amorphous carbon in the chemically treated sample before the welding process, the nanostructured powder presented the lowest crystallinity ratio among the specimens that suffered the thermal treatment, which indicates that the damage caused by the processes mentioned above may have helped the carbide formation, as chromium has a high affinity with carbon^63^.

**Figure 9:**
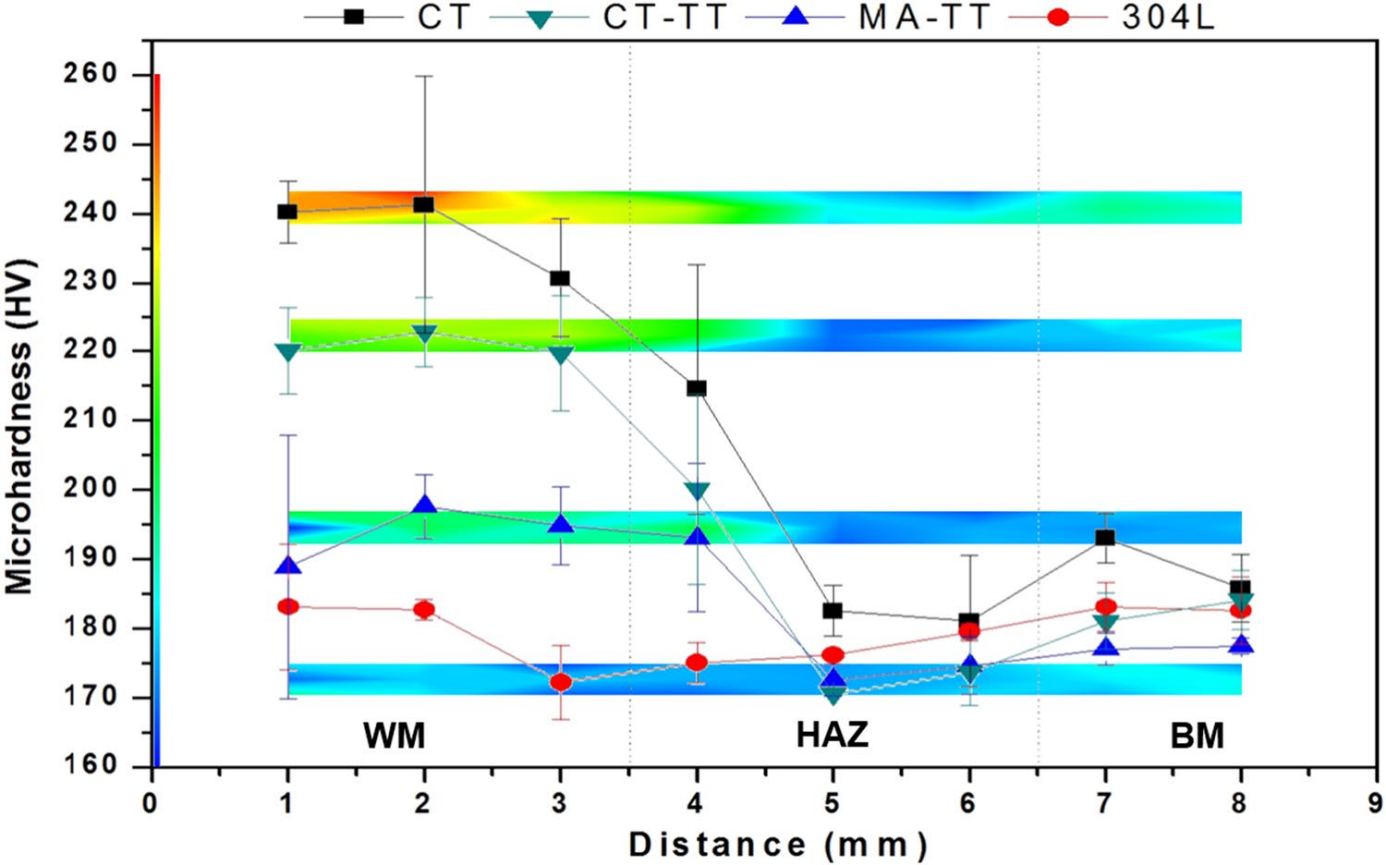
Microhardness mapping of the cross-section of the welded joints with no carbon nanotubes (red circle) and after the processes of MA (blue triangle), CT (black square), and CT-TT (green inverted triangle). Microhardness average distribution of the analyzed samples and its respective standard deviation.

Figure 9 presents the Vickers microhardness average and mapping of the cross-section of the welded joints, highlighting the Weld Metal, the Heat Affected Zone, and Base Metal. By examining the former, there was a considerable improvement in the hardness values throughout the weald bead. If compared to the 304LSS sample, the MA_TT_, CT_TT_, and CT samples increased by an average of up to 8.13%, 22%, and 32.05%, respectively. In addition, some indentations detected a rise of more than 43.50% in the CT one, possibly due to the carbide formation that was detected in the microstructural analysis.

It is possible to observe through SEM analysis that, in the nanostructured composite with a sample submitted to the CT, the grains that compose its microstructure are considerably more prominent than those that were submitted to Mechanical Alloying. This was probably due to the migration of chromium to grain contours, pointed by the EDS, possibly forming carbides that helped to delimit their growth. Because of this, the aforementioned increase in microhardness of the CT sample was possible, if compared to the 304LSS one.

Even though the ACD and ID/IG results regarding the nanostructured powder indicated that the CT_TT_ was the fittest to provide the most expressive hardness increase, since there was neither amorphous carbon nor a high level of defects in the sample, it was not the case. The CT specimen turned out to be the most indicated one to deliver the best mechanical response as a nanostructured composite. This was due to the aggregation state with the metal matrix and its optimized dispersion, as both the XRD and Raman analysis indicated, and the welding process that delivered a Thermal Treatment itself because of the elevated temperatures of the GTAW, which lead the CT sample to a state close to the CT_TT_ one.

## Conclusions

The results indicate that the core weld manufactured with Chemical Treatment using ${\mathrm{H}}_{2}{\mathrm{O}}_{2}$ was the best route to produce a 304LSS-CNT composite via arc welding. As the powder analysis showed, the CT specimen presented the best attachment and dispersion of the CNT throughout the surface of the metal matrix, which led to better utilization of the high surface area of the nanotubes and its subsequent incorporation of carbon into the weld pool. Therefore, the CT route resulted in the best mechanical response when incorporated in cladding, achieving a remarkable hardness increase up to 40%, if compared to a 304LSS one. It represents an outstanding improvement in the manufacturing of nanostructured composites, as the CT is considerably less time consuming than the MA procedure. Its application and mechanical response in surface weldings will be further discussed in future papers.

## Experimental procedure

### Materials

The Multiwalled Carbon Nanotubes used in this paper were produced via Chemical vapor deposition with a 95.7% purity; the 304LSS powder, with a grain size of approximately 44 ± 5 μm. Tables 1 and 2 presents the chemical composition of both the weld joint and metal matrix and its respective mechanical properties.

**Table 1:** Chemical composition of the metal matrix.

Type	Fe	C	Cr	Ni	Mn	Si	S
AISI 304L	64.9–74	≤ 0.03	18–20	8–11.5	≤ 2.00	≤ 1.00	0.030

**Table 2:** Mechanical properties of the metal matrix and MWCNT.

Type	Ultimate strength (MPa)	Yield strength (MPa)	Elongation at break	Vickers microhardness (HV)
AISI 304L	564	210	58%	159
MWCNT	100,000	1,280,000	–	–

### Nanostructured powder routes

To establish the manufacturing route of the 304LSS-CNT nanostructured composite that presents both the better dispersion and aggregation state of the MWCNTs in the metal matrix, therefore assuring the best mechanical properties, two treatment processes of the mixtures were selected, as they are both the least expensive ones and the most simple to be executed regarding the technical apparatus. The selected methods were the Mechanical Alloying (MA), and a Chemical Treatment (CT) with Hydrogen Peroxide and the schematic of the nanostructured powder manufacturing are presented in Fig. 1. Before the nanocomposite powder submission into both routes, the 304LSS metal matrix and 5 wt% MWCNTs (sample 1) were inserted in an ultrasonic bath (55 kHz, 120 W) together with 40 ml of isopropyl alcohol for 10 min to distribute the nanotubes in the metal matrix better, and also to evaporate amorphous carbons; then, the mixture was dried for 30 min at 350 °C (sample 2)^64–66^.

For the Mechanical Alloying route, the mixture was inserted in a planetary ball mill with 30:1 ball-to-powder ratio and then subjected to a 200-rpm speed during 60 min (sample 4), avoiding an overheating, the system was stopped every 10 min for the same amount of time. These parameters were defined as the fittest to manufacture the mixture via MA^56^. As for the Chemical Treatment, after 10 min of isopropyl alcohol, a hydrogen peroxide solution (30% H_2_O_2_) was introduced in ultrasonic bathing. The mixture was then dried for 1 h at 130 °C (sample 6). Both the samples submitted to the MA and the CT routes were inserted in a furnace at 350 °C for 1 h to perform a Thermal Treatment (TT), aiming to eliminate amorphous carbon (samples 3 and 5, respectively)^53^. The samples were analyzed before and after the heating process. MWCNT as-received and TT also were studied.

### Powder characterization

To evaluate the aggregation state of the MWCNTs in the metal matrix and its crystallinity aspects, a Raman spectroscopy Jovin Ivon, model T64000 was used, conducted with 532 nm wavelength; to validate the results, three analysis in each sample were performed. As for the crystal lattice of the nanostructured powder, an X-ray diffraction (XRD) Bruker, model 8 Advance with Bragg-Brentane geometry, Lyns Eye detector, Cu tube, Cu radiation (K α1 = 1.540598) and Ni filter Kβ was necessary. A scanning electron microscope (SEM) VEGA3 TESCAN with a beam acceleration voltage of 20 kV characterized the nanomaterial dispersion in the metal matrix powder.

### Welding procedure

The nanostructured powders served as flux in a tubular rod (2 and 1.45 mm of outer and inner wire, respectively) of 304LSS, as described by Loayza et al.^56^, in a simple deposition weld beam over 304LSS plates. Another tubular rod solely used 304LSS metal matrix powder as flux for comparative analysis. The process was P-GTAM, using pure Argon (Ar) as shielding gas. The parameters used in the manual welding process were 14 V voltage, 130 A peak current, 80 A base current, 0.5 s peak time, 1 s base time, 3.33 mm s^−1^ welding velocity, 0.24 kJ mm^−1^ heat input, a tungsten electrode (3 mm), and 5 mm stick out. The nanostructured cored wire was incorporated directly in the weld pool to avoid direct contact with high temperatures of the electric arc.

### Nanocomposite characterization

The simple deposition welds were characterized via Scanning electron microscope, using Backscattered-Electron (BSE) and Energy-Dispersive X-ray Spectroscopy (EDS) to evaluate its microstructural features. Microhardness (HV0.3) was performed to analyze the mechanical properties of the specimens, under the standard ASTM E384-17.

## Supplementary information

Supplementary Information.
