# Supplementary material for: Stainless steel weld metal enhanced with carbon nanotubes
Source: Sci Rep. 2020 Oct 21;10:17977. doi: 10.1038/s41598-020-75136-z (PMC7578026; doi:10.1038/s41598-020-75136-z)
Supplement: Supplementary file 1 — Supplementary Information. [file 41598_2020_75136_MOESM1_ESM.docx]

**STAINLESS STEEL WELD METAL ENHANCED WITH CARBON NANOTUBES**

***Borges D. J. A.^a^; Cardoso D. C. S.^a^; Braga E. M.^a, b^; Castro A. A. F.^a^; Dos Reis M. A. L.^b, c^; Loayza C. R.^b^**

**a** Programa de Pós-graduação em Engenharia Mecânica (PPGEM/UFPA), Universidade Federal do Pará, 66075-110, Belém PA, Brazil.

**b** Programa de Pós-Graduação em Engenharia de Recursos Naturais da Amazônia (PRODERNA/ITEC), Universidade Federal do Pará, 66075-110, Belém PA, Brazil.

**c** Faculdade de Ciências Exatas e Tecnologia, Universidade Federal do Pará, 68440-000, Abaetetuba PA, Brazil.

*Correspondence to “diego.jborges70@gmail.com”

**Supplementary material**


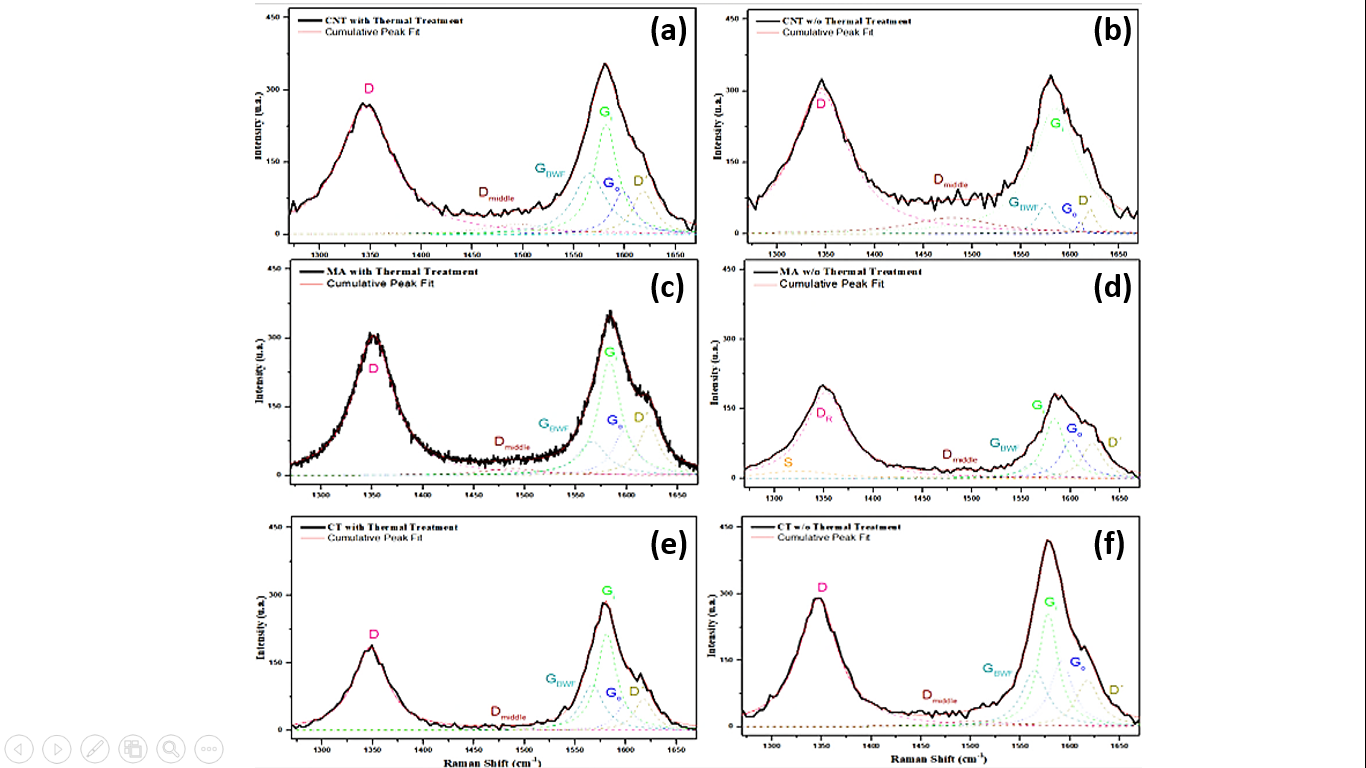


S 1 Raman spectroscopy features of the D-band and G-band to a) CNT_TT_, b) CNT, c) MA_TT_, d) CNT_,_ e) CT_TT_, and f) Chemically treated nanostructured powders. Showing the deconvoluted sub band peaks S, D_l_, D_r_, D_middle_, G_BWF_, G_i_, G_o_, D´.


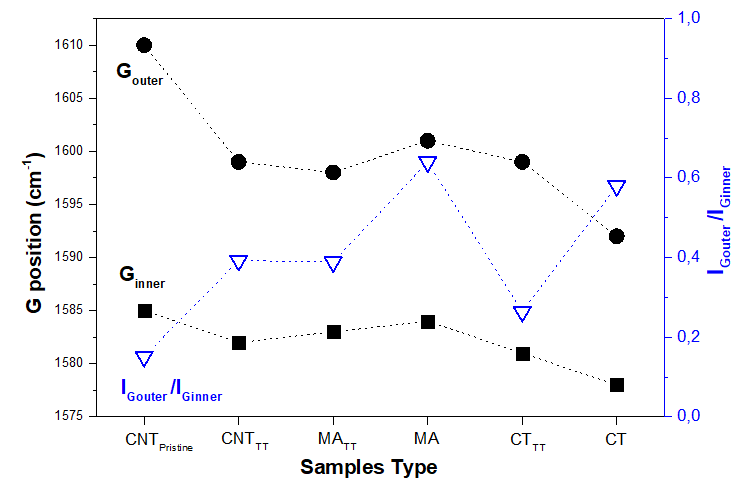


**(a)**


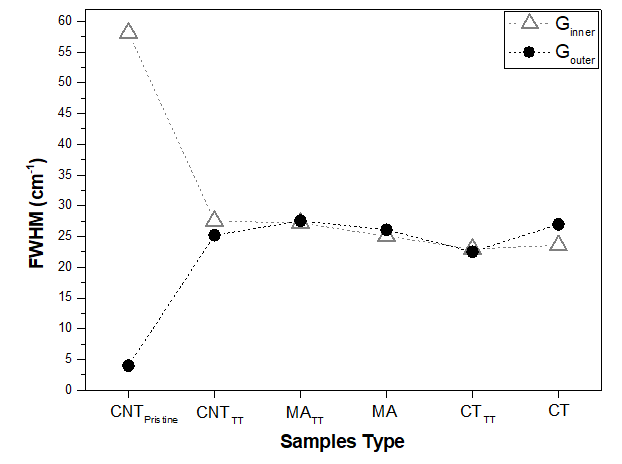


**(b)**

S 2 The (a) presents the *I_Gouter_/I_Ginner_* ratio and the G position. (b) FWHM as a function of treatment received by the CNT.


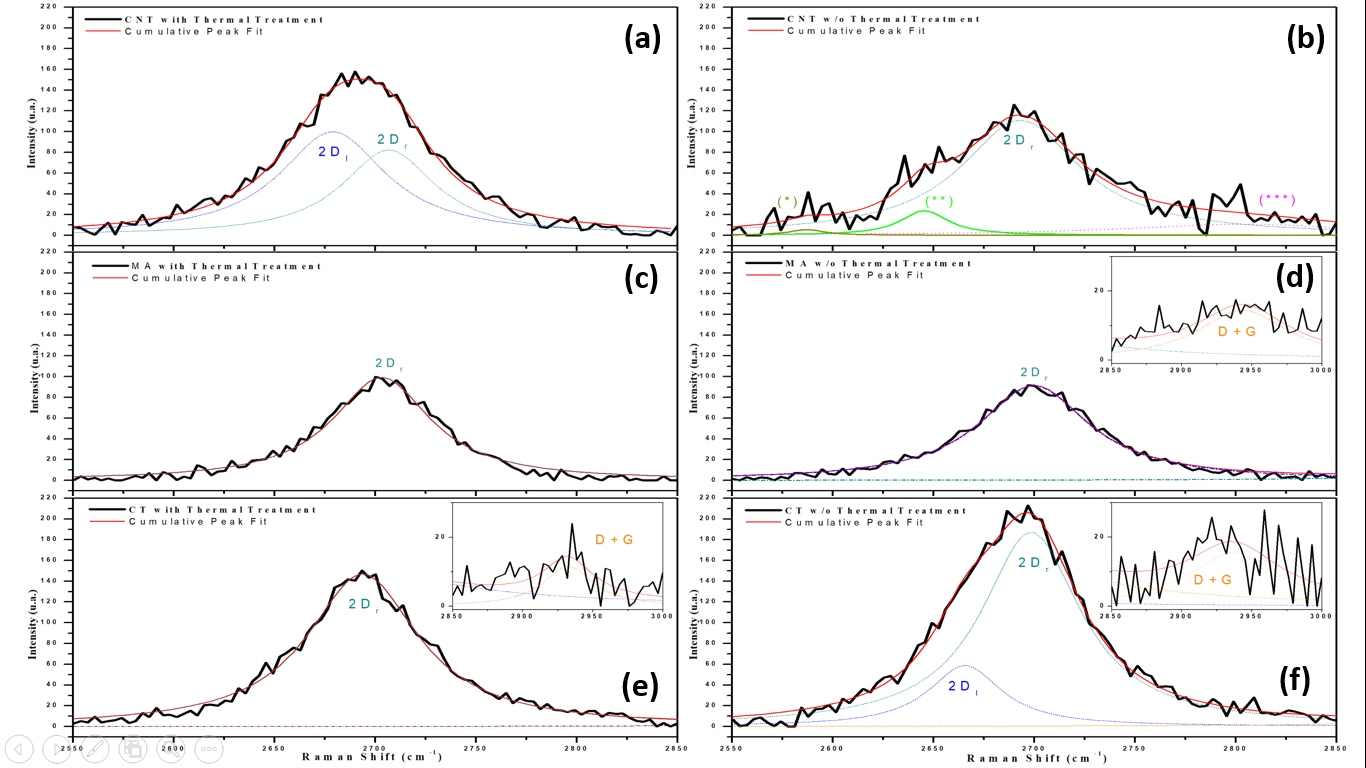


S 3 Raman spectroscopy features of the 2D and D+G sub-bands to a) CNT_TT_, b) CNT, c) MA_TT_, d) CNT_,_ e) CT_TT_ and f) Chemically treated nanostructured powders. Showing the deconvoluted sub band peaks S, D_l_, D_r_, D_middle_, G_BWF_, G_i_, G_o_, D´. (*), (**), and (***) are observed only in the pristine CNT.


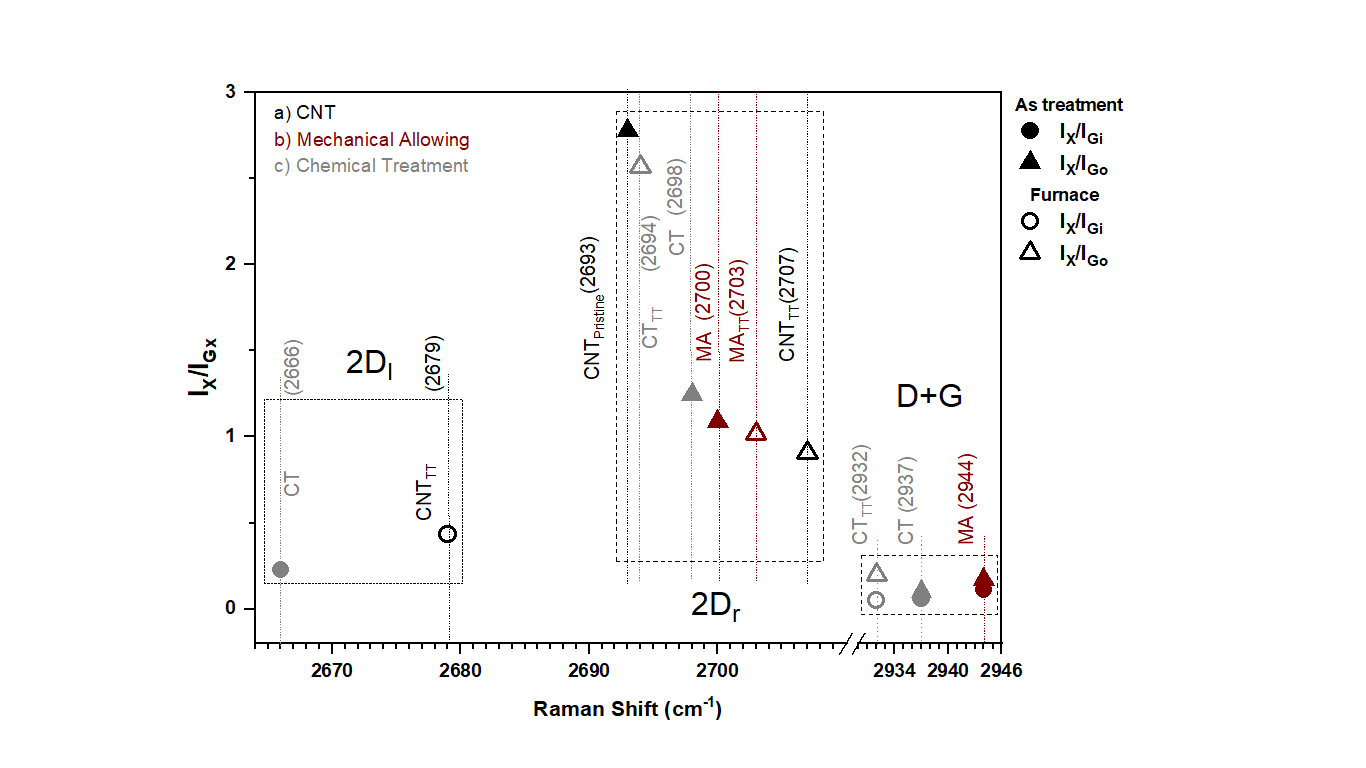


S 4 Intensity ratios of peaks: second order (2D_l_, 2D_r_ and D+G) where the I_x_ corresponds to intensities for each Raman band to the various samples.

S 5 Tables to various samples showing Center, Amplitude, FWHM, Analytic Area, and Integral Area.

|  | **CNT with TT** | | | | | |
| --- | --- | --- | --- | --- | --- | --- |
| **Sub-band** | | **Center (cm^-1^)** | **Amplitude**  **(u. a.)** | **FWHM (cm^-1^)** | **Analytic Area (u.a.)** | **Integral Area**  **(u. a.)** |
| **S** | | 1164 | 9 | 79 | 1074 | 986 |
| **D** | | 1346 | 264 | 65 | 27010 | 25834 |
| **D*_middle_*** | | 1496 | 21 | 76 | 2515 | 2398 |
| **G*_BWF_*** | | 1566 | 128 | 38 | 7650 | 7468 |
| **G*_inner_*** | | 1582 | 231 | 28 | 9984 | 9811 |
| **G*_outer_*** | | 1599 | 91 | 25 | 3589 | 3531 |
| **D´** | | 1618 | 91 | 25 | 3620 | 3561 |
| **2D_l_** | | 2679 | 100 | 64 | 10110 | 9095 |
| **2D_r_** | | 2707 | 82 | 56 | 7290 | 6603 |

|  | **CNT_Pristine_** | | | | | |
| --- | --- | --- | --- | --- | --- | --- |
| **Sub-band** | | **Center (cm^-1^)** | **Amplitude**  **(u. a.)** | **FWHM (cm^-1^)** | **Analytic Area (u.a.)** | **Integral Area**  **(u. a.)** |
| **D** | | 1345 | 296 | 70 | 32429 | 30534 |
| **D*_middle_*** | | 1479 | 34 | 104 | 5490 | 5083 |
| **G*_BWF_*** | | 1576 | 64 | 20 | 2012 | 1983 |
| **G*_inner_*** | | 1585 | 264 | 58 | 24117 | 23095 |
| **G*_outer_*** | | 1610 | 40 | 4 | 249 | 248 |
| **D´** | | 1621 | 53 | 13 | 1068 | 1057 |
| **DDl*** | | 2587 | 5 | 22 | 187 | 169 |
| **2D**** | | 2646 | 24 | 28 | 1068 | 1002 |
| **2D_r_** | | 2693 | 111 | 72 | 12596 | 11012 |
| **DGl***** | | 2803 | 11 | 142 | 2402 | 1800 |

|  | **MA with TT** | | | | | |
| --- | --- | --- | --- | --- | --- | --- |
| **Sub-band** | | **Center (cm^-1^)** | **Amplitude**  **(u. a.)** | **FWHM (cm^-1^)** | **Analytic Area (u.a.)** | **Integral Area**  **(u. a.)** |
| **D** | | 1352 | 303 | 48 | 23028 | 22018 |
| **D*_middle_*** | | 1489 | 15 | 72 | 1649 | 1550 |
| **G*_BWF_*** | | 1566 | 74 | 35 | 4074 | 3945 |
| **G*_inner_*** | | 1583 | 250 | 27 | 10676 | 10407 |
| **G*_outer_*** | | 1598 | 97 | 28 | 4211 | 4100 |
| **D´** | | 1622 | 110 | 26 | 4409 | 4295 |
| **2D_r_** | | 2703 | 99 | 59 | 9237 | 8431 |

|  | **MA** | | | | | |
| --- | --- | --- | --- | --- | --- | --- |
| **Sub-band** | | **Center (cm^-1^)** | **Amplitude**  **(u. a.)** | **FWHM (cm^-1^)** | **Analytic Area (u.a.)** | **Integral Area**  **(u. a.)** |
| **S** | | 1323 | 16 | 82 | 2090 | 1975 |
| **D** | | 1352 | 186 | 51 | 14961 | 14466 |
| **D*_middle_*** | | 1492 | 7 | 63 | 702 | 677 |
| **G*_BWF_*** | | 1561 | 33 | 27 | 1388 | 1367 |
| **G*_inner_*** | | 1584 | 131 | 25 | 5141 | 5068 |
| **G*_outer_*** | | 1601 | 84 | 26 | 3427 | 3377 |
| **D´** | | 1622 | 77 | 24 | 2972 | 2930 |
| **2D_r_** | | 2700 | 91 | 66 | 9539 | 8865 |
| **D+G** | | 2944 | 14 | 79 | 1793 | 1628 |

|  | **CT with TT** | | | | | |
| --- | --- | --- | --- | --- | --- | --- |
| **Sub-band** | | **Center (cm^-1^)** | **Amplitude**  **(u. a.)** | **FWHM (cm^-1^)** | **Analytic Area (u.a.)** | **Integral Area**  **(u. a.)** |
| **D_l_** | | 1348 | 184 | 41 | 11849 | 11471 |
| **D*_middle_*** | | - | - | - | - | - |
| **G*_BWF_*** | | 1567 | 102 | 26 | 4244 | 4165 |
| **G*_inner_*** | | 1581 | 217 | 23 | 7827 | 7699 |
| **G*_outer_*** | | 1599 | 57 | 23 | 2022 | 1989 |
| **D´** | | 1618 | 71 | 21 | 2381 | 2343 |
| **2D_r_** | | 2694 | 147 | 67 | 15525 | 14230 |
| **D+G** | | 2932 | 12 | 43 | 780 | 728 |

|  | **CT** | | | | | |
| --- | --- | --- | --- | --- | --- | --- |
| **Sub-band** | | **Center (cm^-1^)** | **Amplitude**  **(u. a.)** | **FWHM (cm^-1^)** | **Analytic Area (u.a.)** | **Integral Area**  **(u. a.)** |
| **S** | |  |  |  |  |  |
| **D** | | 1347 | 289 | 48 | 21841 | 20960 |
| **D*_middle_*** | | 1505 | 13 | 82 | 1713 | 1610 |
| **G*_BWF_*** | | 1564 | 129 | 31 | 6269 | 6124 |
| **G*_inner_*** | | 1578 | 259 | 24 | 9580 | 9409 |
| **G*_outer_*** | | 1592 | 150 | 27 | 6363 | 6231 |
| **D´** | | 1618 | 106 | 28 | 4650 | 4548 |
| **2D_l_** | | 2666 | 59 | 44 | 4059 | 3860 |
| **2D_r_** | | 2698 | 187 | 60 | 17654 | 16541 |
| **D+G** | | 2937 | 15 | 78 | 1884 | 1685 |


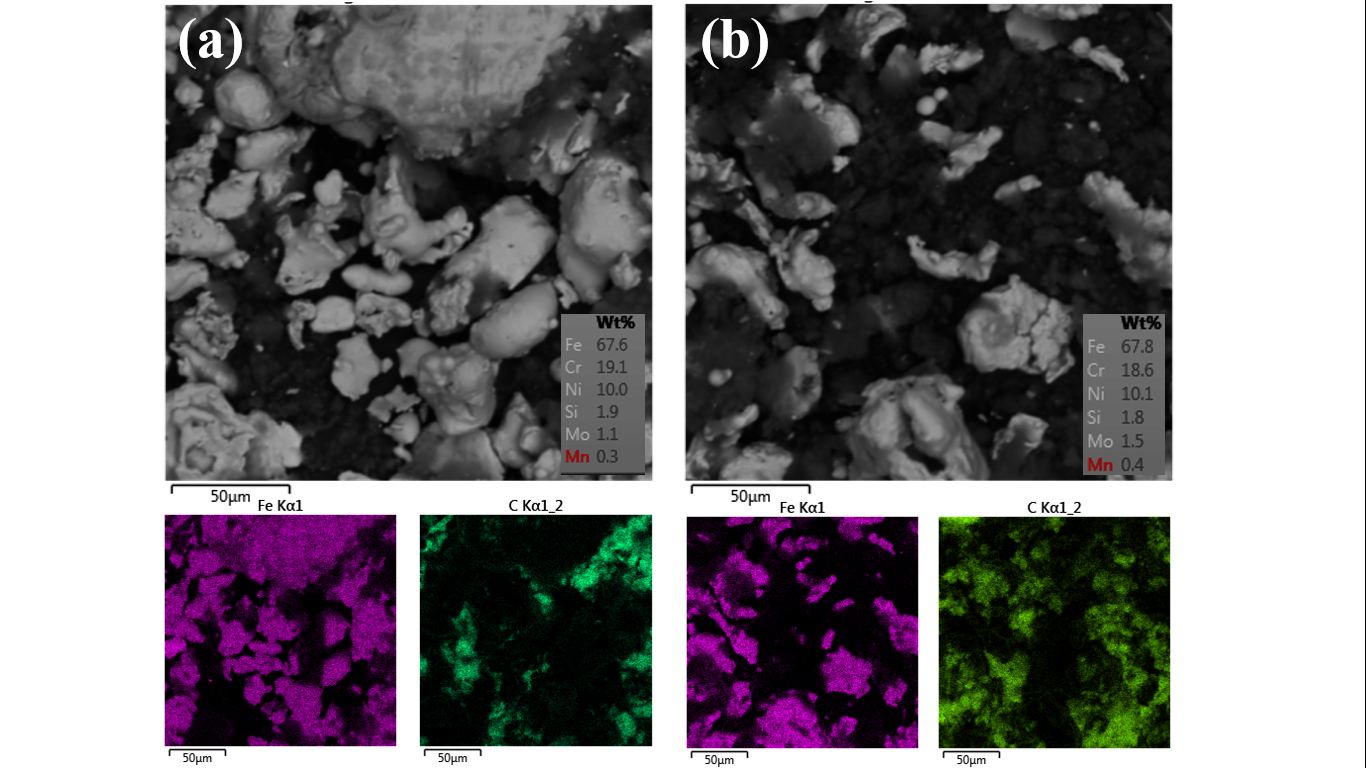

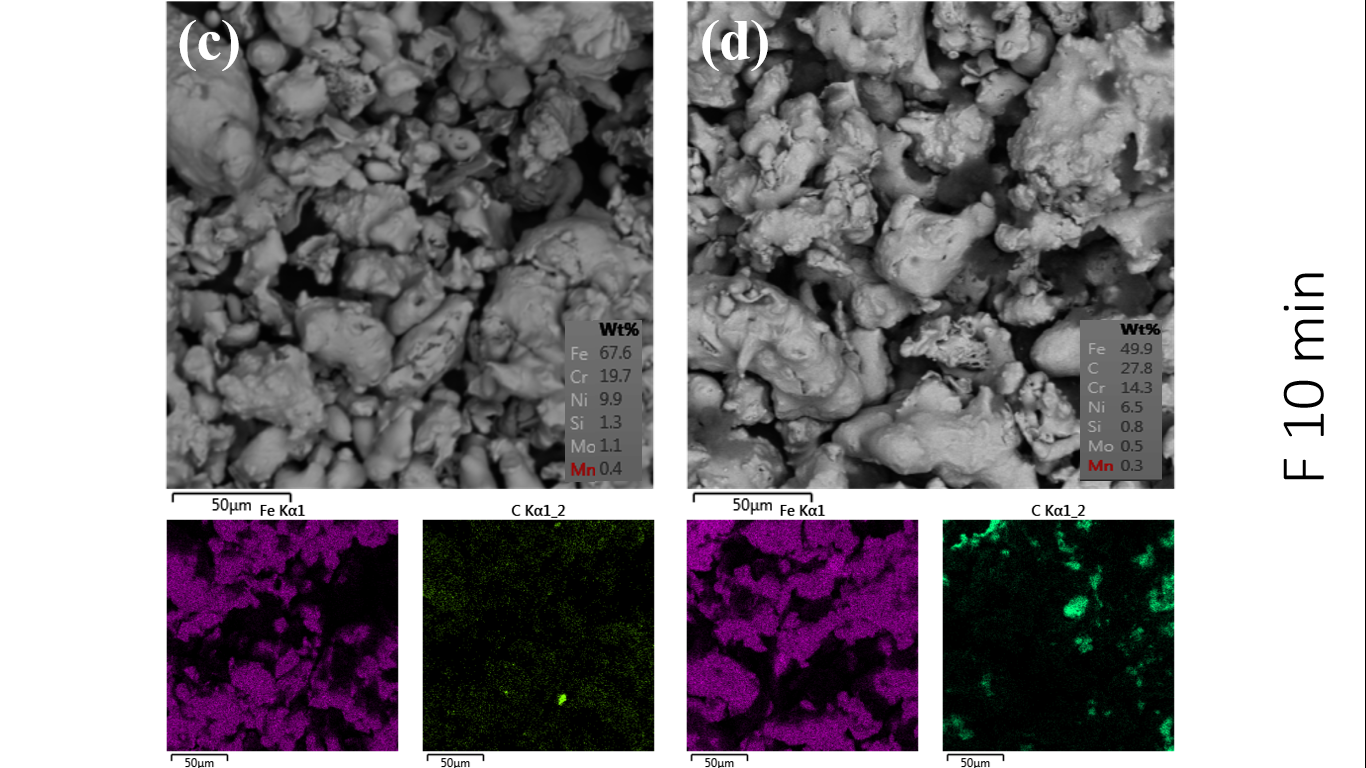


S 6 **SEM images of the a) after ultrasonicated b) Mechanically alloyed and Chemically treated nanostructured powders c) with and d) without TT** with high magnifications. Here is shown that the sample with CT_TT_ has not a uniform distribution of the MWCNT; the distribution is better in the other samples, indicating a good interaction and wettability with the MM.


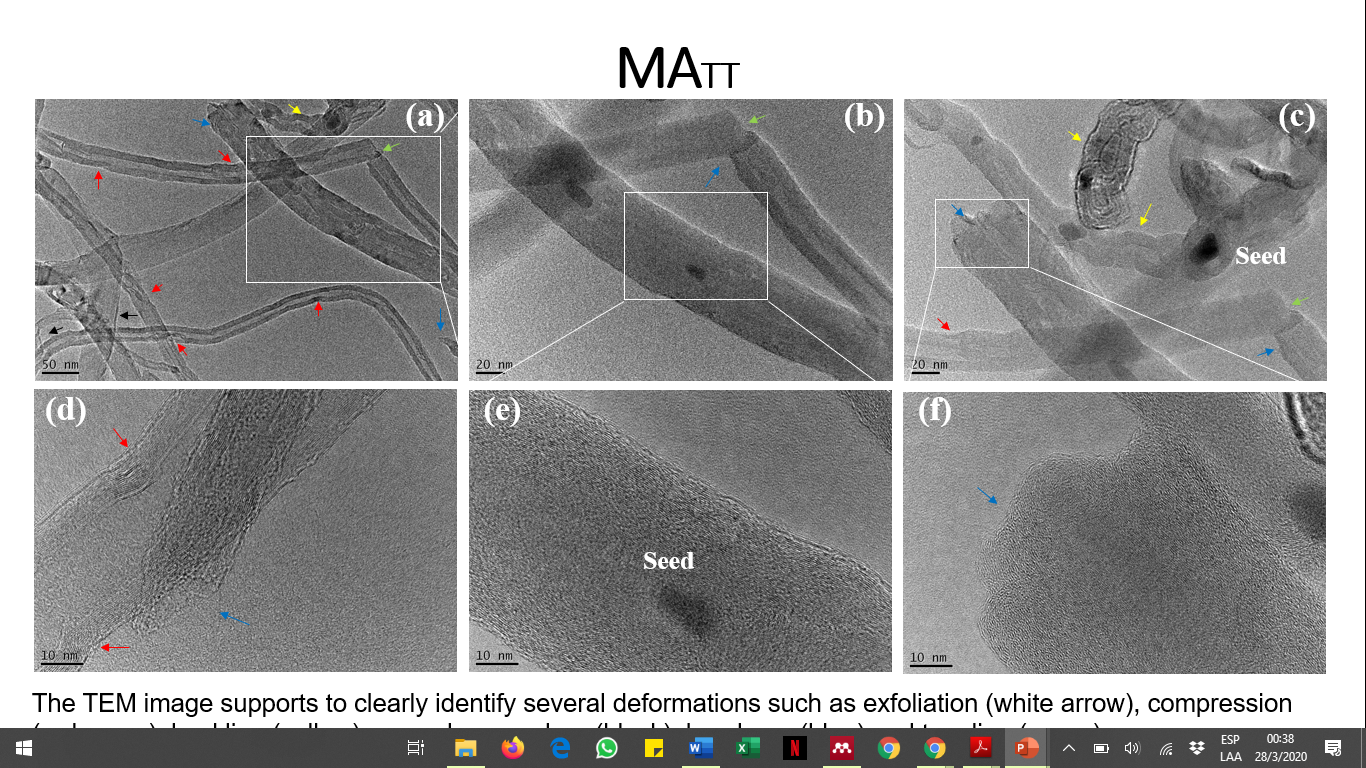


S 7 The TEM image supports to clearly identify several deformations such as exfoliation (white arrow), compression (red arrow), buckling (yellow), defects (black), breakage (blue) and tangling (green) to the MA_TT_. The pictures from a) to f) identify different regions of the same specimen. There is low amorphous carbon, but the MWCNT are shortened and break, evidencing many tips and defects.


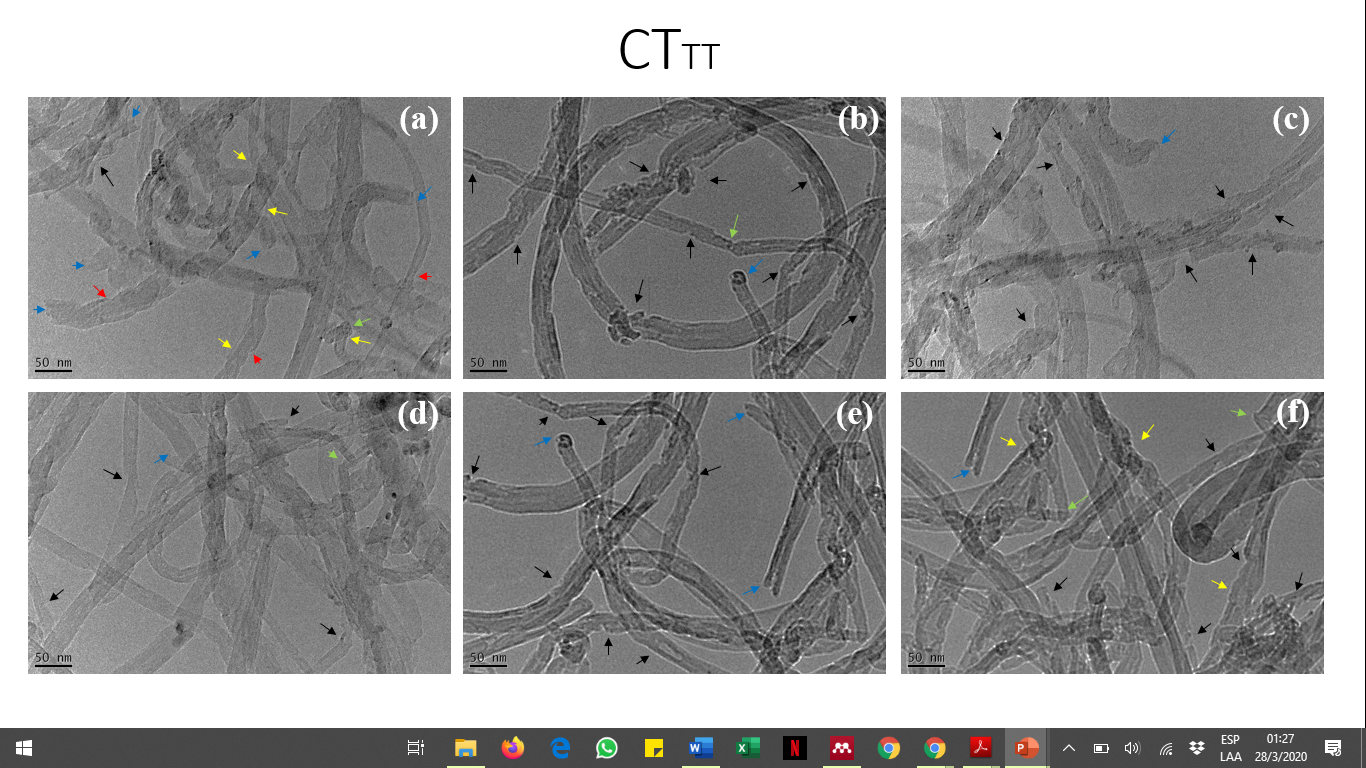


S 8 The TEM image supports to clearly identify several deformations such as exfoliation (white arrow), compression (red arrow), buckling (yellow), defects (black), breakage (blue) and tangling (green) to the CT_TT_. The pictures from a) to f) identify different regions of the same specimen. There is no evidence of amorphous carbon, but the MWCNT are damaged and break, evidencing many tips and defects. This could indicate that the CT process act as point defects concentration, that severe attack the walls with the thermal treatment.


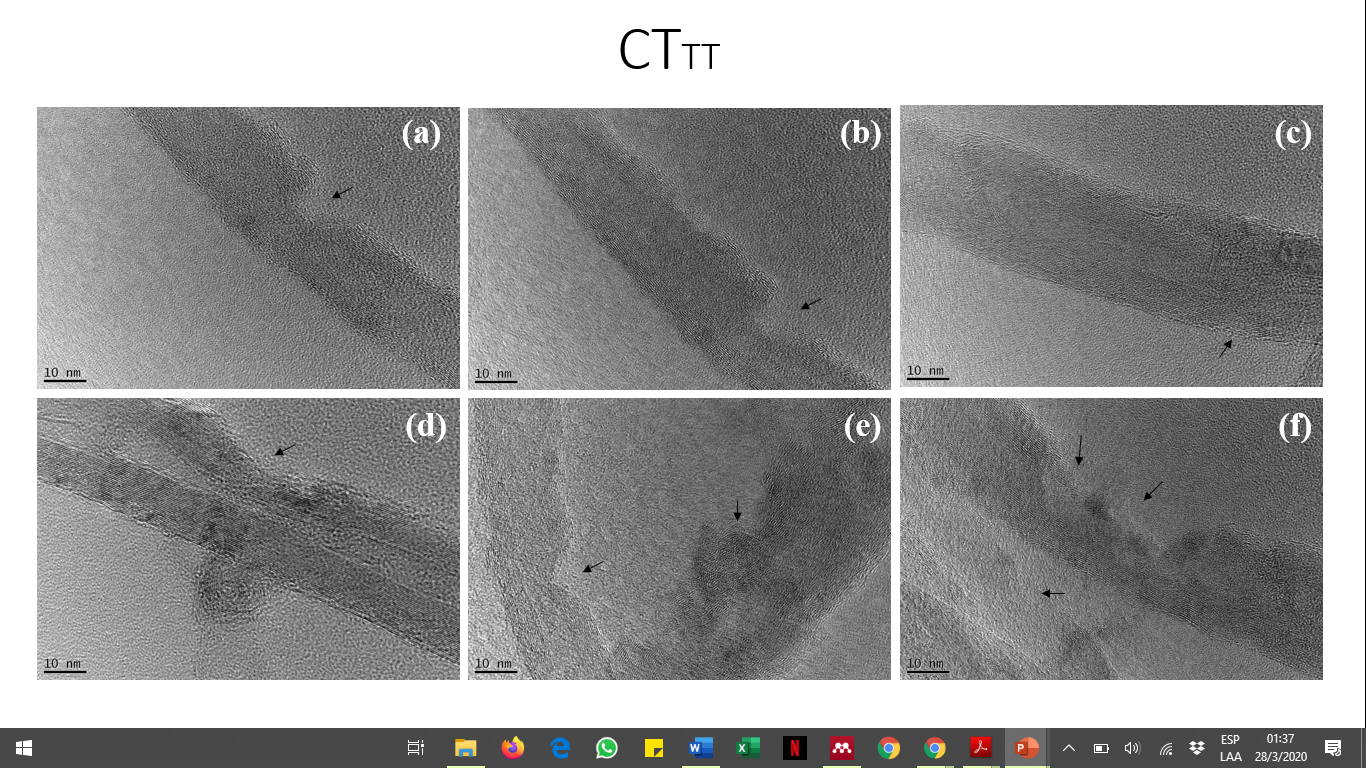


S 9 The TEM image supports to clearly identify several deformations such as defects (black arrows) to the CT_TT_. The pictures from a) to f) identify different regions of the same specimen. There is no evidence of amorphous carbon, but the MWCNT are damaged significantly, showing many defects.


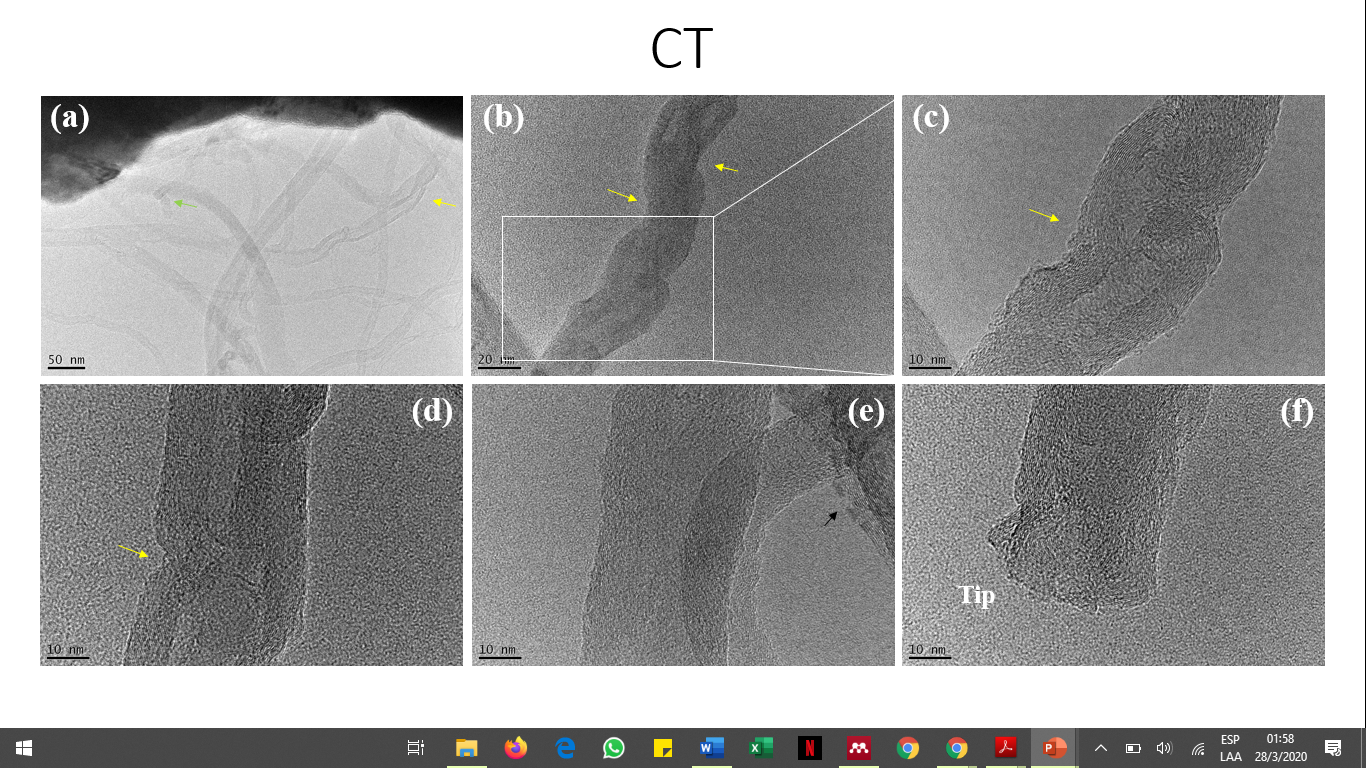


S 10 The TEM image supports to clearly identify several deformations such as compression buckling (yellow arrows), defects (black), and tangling (green) to the CT. The pictures from a) to f) identify different regions of the same specimen. It did not show amorphous carbon, low defects, and good surface structure, indicating that the hexagonal lattice is maintained.
